# Imaging Immune Cells Using Fc Domain Probes in Mouse Cancer Xenograft Models

**DOI:** 10.3390/cancers14020300

**Published:** 2022-01-08

**Authors:** Wendy Bernhard, Kris Barreto, Ayman El-Sayed, John DeCoteau, C. Ronald Geyer

**Affiliations:** Translational Research Cluster, Department of Pathology and Laboratory Medicine, University of Saskatchewan, Saskatoon, SK S7N 5E5, Canada; wendylynnette@gmail.com (W.B.); dr.barreto@gmail.com (K.B.); meayman@gmail.com (A.E.-S.); john.decoteau@usask.ca (J.D.)

**Keywords:** Fc domain, near-infrared fluorescence imaging, IRDye800CW, immune cell tracking, tumor infiltration

## Abstract

**Simple Summary:**

The immune system responds to abnormal cell growth by sending immune cells to kill them. The immune cell response is very important since it can usually stop abnormal cells from growing and spreading. Immuno-therapeutics used to treat cancer require help from the immune system to be effective. A biopsy is typically performed to determine the therapeutic efficacy of cancer treatment which is invasive and difficult. A simpler and less invasive way to monitor therapeutic efficacy is needed. Here, we show a molecule that can be used as an imaging agent to determine immune cell recruitment to tumors.

**Abstract:**

Tracking immune responses is complex due to the mixture of cell types, variability in cell populations, and the dynamic environment. Tissue biopsies and blood analysis can identify infiltrating and circulating immune cells; however, due to the dynamic nature of the immune response, these are prone to sampling errors. Non-invasive targeted molecular imaging provides a method to monitor immune response, which has advantages of providing whole-body images, being non-invasive, and allowing longitudinal monitoring. Three non-specific Fc-containing proteins were labeled with near-infrared dye IRDye800CW and used as imaging probes to assess tumor-infiltrating immune cells in FaDu and A-431 xenograft models. We showed that Fc domains localize to tumors and are visible by fluorescent imaging. This tumor localization appears to be based on binding tumor-associated immune cells and some xenografts showed higher fluorescent signals than others. The Fc domain alone bound to different human immune cell types. The Fc domain can be a valuable research tool to study innate immune response.

## 1. Introduction

The innate immune system is the first to respond to infection or injury with recruitment of granulocytes and then monocytes [1]. This sets off a cascade of signals amplifying the acute inflammatory response. Since the response is self-limiting, inflammation is usually resolved after a few days; however, if factors that mediate resolution are not in place, chronic infection can lead to inflammatory diseases such as Crohn’s disease, psoriasis, type II diabetes, atherosclerosis, and cancer [1]. Cancers develop from transformed cells that have managed to evade the immune system and gain growth advantage over normal cells [2]. In doing this, cancer cells use immune cells such as monocytes and neutrophils to aid in their development [3]. Having a way to identify the innate immune cell recruitment would be useful for disease diagnosis and treatment of cancer. Monocytes, for example, have cancer-promoting properties and are associated with therapy-resistant cancer microenvironments [3]. Depending on the cancer, some therapies including doxorubicin, paclitaxel, and radiotherapy have been shown to increase monocyte recruitment and promote relapse, metastasis, and treatment resistance. By monitoring monocytes during these types of treatments, therapeutic success could be determined early on in treatment to address this before the cancer has metastasized or become resistant to treatment.

Current techniques used to monitor immune cells are invasive and prone to error. In solid tumors, a biopsy is collected, and histopathology is used to determine immune cell infiltration [4,5]. This is equivalent to taking a ‘snap shot’ of the tumor, which is dynamic and can change over time. Non-invasive molecular imaging of immune cell infiltration is less evasive and, after injection with the imaging agent, multiple imaging time points can be obtained if necessary [6]. In research, this is useful since it reduces on the number of animals required for an experiment. In the clinic, this can lead to identification of immune infiltration and provides a way to monitor therapeutic efficacy.

Molecular imaging agents have been developed for this purpose and show promise [7,8,9,10,11,12]. [^18^F]-FDG, for example, is the gold-standard molecular imaging agent currently used to image cancer and has also been used to image immune responses using PET [6]. [^18^F]-FDG is taken up by most tumors but is also taken up by myeloid cells. This can make it difficult to distinguish between inflammation in the tumor and cancer cells in some cases [13,14,15]. Some cancer therapies, specifically those targeting the immune response or ionizing radiation, can increase tumor infiltration by immune cells [16,17]. Follow-up [^18^F]-FDG scans showing an increase in uptake may actually be from inflammation and not from additional tumor growth [6,15]. Targeted imaging probes are needed that directly image immune cells and are able to distinguish between tumor cells and inflammation. Here, we explored whether Fc domains could be used as imaging probes to characterize immune cell infiltration in tumors.

## 2. Materials and Methods

### 2.1. Cloning and Expression of VHH-Fc and Fc Domain

The human Fc domain was cloned using the pFUSEss-CHIg-hG1 vector (Invivogen) by removing the DNA sequence after the IL2 signal sequence (including the CH1 domain) up to the CH2 domain. The Fc contains the IL2 signal sequence, CH2, and CH3, which gives the following protein sequence: MYRMQLLSCIALSLALVTNSEPKSCDKTHTCPPCPAPELLGGPSVFLFPPKPKDTLMISRTPEVTCVVVDVSHEDPEV-KFNWYVDGVEVHNAKTKPREEQYNSTYRVVSVLTVL-HQDWLNGKEYKCKVSNKA-LPAPIEKTISKAKGQPREPQVYTLPPSREEMTKNQVSL-TCLVKGFYPSDIAVEWESNGQPENNYKTTPPVLDSDGSFFLYSKLTVDKSRWQQG-NVFSCSVMEALHNHYTQKSLSLSPGK. The VHH-Fc targets FaDu and MDA-MB-231 cells and contains a VHH linked to the Fc described above. The IgG and scFv-Fc that bind maltose binding protein were sub-cloned from a Fab fragment as previously described [18,19].

For protein expression, 1 µg of the total DNA of the IgG-, scFv-Fc-, VHH-Fc-, or Fc-expressing plasmids per milliliter of cells was transfected into Expi293 cells (ThermoFisher) and expressed according to the manufacturer’s instructions. Briefly, the enhancers were added for one week. Cells and supernatant were separated by centrifugation at 1000× *g* for 5 min. The supernatant was collected and filtered through a 0.45 µm filter unit (Millipore). Antibodies and fragments were purified from the supernatant using an AKTAprime plus (GE Healthcare Life Sciences) with a HiTrap MabSelect SuRe column (GE Healthcare Life Sciences) according to the manufacturer’s instructions. The purified protein was sterilized using a 2 µm filter unit and dialyzed with 1 L of PBS twice using 7 MWCO dialysis tubing. Purified protein was analyzed by microcapillary electrophoresis using an Agilent Bioanalyzer 2100 according to the manufacturer’s instructions.

### 2.2. Tissue Culture

The Expi293 cell line was purchased from Thermofisher Scientific (Waltham, MA, USA). SEM cells were purchased from (DSMZ). All other cell lines were purchased from ATCC. Mv4-11, SEM, and K562 cells were cultured in 90% Iscove’s Modified Dulbecco’s Medium (IMDM) and 10% fetal bovine serum (FBS). Jurkat, A-431, and DLD-1 cells were cultured in 90% Roswell Park Memorial Institute medium (RPMI) and 10% FBS. FaDu cells were cultured in 90% Minimum Essential Medium (MEM/EBSS) with Earle’s balanced salts and 10% FBS. MDA-MB-231 cells were cultured in 90% Dulbecco’s Modified Eagle Medium (DMEM) and 10% FBS.

### 2.3. Protein Labeling and Flow Cytometry

In total, 1 mg of IgG, scFv-Fc, VHH-Fc, or Fc was labeled with IRDye680RD NHS ester (LI-COR Biosciences, Lincoln, NE, USA) for flow cytometry or IRDye800CW NHS ester (LI-COR Biosciences) for optical imaging experiments, according to the manufacturer’s instructions. Briefly, the Fc and IgG and fragments were labeled at a 1:3 protein:dye ratio in PBS at 4 °C overnight on a rotator. Excess dye was removed using a Zeba spin desalting column 7 MWCO, according to the manufacturer’s instructions (Thermofisher Scientific).

Flow cytometry was performed on cancer cell lines mv4-11 (macrophage Jurkat (T-cell), SEM (B-cell), K562 (erythroleukemia), HL-60 (promyeloblast), A-431(epithelial), FaDu (epithelial), and MDA-MB-231 (epithelial). In total, 1 × 10^5^ cells were collected for each titration point. Cells were washed with PBSF (phosphate-buffered saline and 2% FBS) and suspended in PBSF. IRDye680RD-labeled IgG, scFv-Fc and Fc (0–2 µM) were titrated with 1 × 10^5^ cells, incubated at room temperature for 30 min and then on ice for 15 min. Cells were washed with PBSF and analyzed on a Beckman Coulter Gallios flow cytometer. The data were analyzed using FlowJo version 10.5.3 and GraphPad Prism version 6. Mean fluorescent intensity (MFI) values were normalized and analyzed by a non-linear regression curve fit to obtain K_Dapp_ values. For single point binding analysis, the unstained population was set to 0.5% in a FL7 (IRDye680RD) positive gate. The percent positive cells in that gate at 100 nM of IRDye680RD-labeled protein were recorded.

Flow cytometry experiments with human peripheral blood were performed by lysing red cells with lysis buffer (150 mM NH_4_Cl, 10 mM NaHCO_3_, and 1.3 mM EDTA) for 10 min at room temperature. Cells were centrifuged at 1000 rpm for 5 min, washed with PBSF, and counted. 3 × 10^6^ cells were stained with immune panel antibodies (CD3, CD4, CD19, CD14, CD8, CD56, CD16, CD7, and CD45) (Beckman Coulter, Brea, CA, USA) with or without IRDye680RD-Fc for 20 min at room temperature. Cells were washed and analyzed on a Beckman Coulter Gallios flow cytometer.

The data were analyzed using Kaluza Flow cytometry analysis software. Cells were gated on singlets by plotting forward scatter (FSC) vs. FSC and on intact cells by plotting FSC vs. side scatter (SSC). Using the intact gate, white blood cells (WBCs) were gated by plotting CD45 vs. SSC. The lymphocyte population was further refined using the WBC gate and plotting FSC vs. SSC. Lymphocytes and myelocytes/monocytes were separated using the WBC gate and plotting CD45 vs. SS. T cells and B cells were separated by using the lymphocyte gate and plotting CD3 vs. CD19. The myelocyte/monocytes gate was separated into monocytes and granulocytes by plotting CD14 vs. CD16. IRDye680RD-Fc (Fc-680) that bound to B cells were analyzed using the B-cell gate and plotting CD19 vs. Fc-680. IRDye680RD-Fc that bound to T cells were analyzed using the T-cell gate and plotting CD3 vs. Fc-680. IRDye680RD-Fc that bound to monocytes were analyzed using the monocyte gate and plotting CD14 vs. Fc-680. IRDye680RD-Fc that bound to granulocytes were analyzed using the granulocyte gate and plotting CD16 vs. Fc-680. Natural killer (NK) cells were separated out from the lymphocyte gate by plotting the CD3- vs. CD7+. From this gate the NK cells were analyzed using the NK gate by plotting CD56 vs. Fc-680.

### 2.4. Mouse Xenograft Models, Imaging, and Xenograft and Bone Marrow Flow Cytometry

All mouse experiments were performed with approval and under the supervision and guidelines of the University of Saskatchewan Animal Care Committee (protocol # 20160112). Female CD-1 nude mice were obtained from Charles River Canada. All mice had ad libitum access to food and water. Mice 6–8 weeks old were used for experiments. For xenografts, ten million cells (FaDu, A-431, or MDA-MB-231) were washed with serum-free media and suspended in 50 µL of serum-free media and 50 µL of Matrigel Matrix (Corning) on ice. The cell mixture was injected subcutaneously into the right hind flank of CD-1 nude female mice. Xenografts were monitored with external calipers until they reached a size of 150–300 mm^3^. Once xenografts reached this size the mice were injected with 0.5 nmoles of IRDye800CW-labeled IgG, scFv-Fc, VHH-Fc, or Fc. The mice were imaged over time using a LI-COR Pearl small animal imaging system. For the Fc blocking experiment 75× (37.5 nmoles) the amount of unlabeled Fc was injected prior to the injection of the VHH-Fc.

Three regions of interest (ROI) were selected from equal sized areas containing the same number of pixels for xenografts, liver, kidney, and contralateral side. The MFI of ROIs were averaged then normalized by labeling ratio. A two-way analysis of variance (ANOVA) with multiple comparisons using GraphPad Prism was used to compare the normalized fluorescent signals and tumor-to-background ratios (TBR) for accumulation of the different proteins and blocking experiment in xenografts. All experiments were performed with at least three biological replicates (*n* ≥ 3) and represent the standard error of the mean (SEM).

Flow cytometry from mouse xenograft and bone marrow cells isolated from mice bearing A-431 and FaDu xenografts was performed 24 h after injection of 0.5 nmols IRDye800CW-Fc. Xenografts were collected and a single-cell suspension was made by shearing tissue with needles in RPMI media. Bone marrow was flushed from the mouse bones with RPMI media. Xenograft and bone marrow cell suspensions were passed through a cell strainer and washed and suspended in a solution of PBS, 0.5% FBS, and 1 mM EDTA. Cells were stained with biotinylated mouse lineage antibodies specific for T cells, B cells, monocytes/macrophages, granulocytes and erythrocytes (CD5, CD11b, CD45R, Anti7-4, Anti-Gr-1 (Ly6G/C, and Anti-Terr-119) (Miltenyi Biotec, Bergisch Gladbach, Germany) and streptavidin-PE secondary antibody or secondary alone and analyzed on a Beckman Coulter Gallios flow cytometer. The data were analyzed using FlowJo version 10.5.3.

## 3. Results

### 3.1. Fc Imaging Probe Expression and Fluorescent Labeling

**Figure 1:**
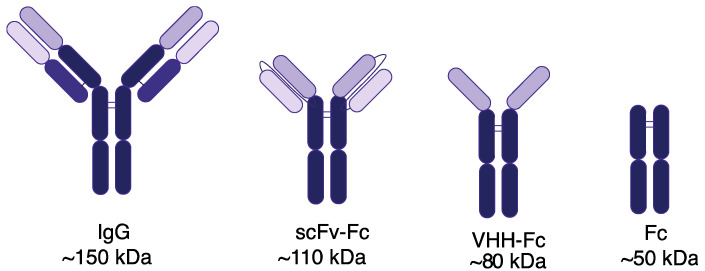
Fc-containing imaging probes. Probes used for this study are an IgG (~150 kDa), a scFv-Fc (~110 kDa), a VHH-Fc (~80 kDa), and an Fc domain (~50 kDa). An IgG is comprised of a light chain and heavy chain. Antigen binding is mediated by complementarity-determining regions found in the variable domains of the light chain (light grey) and heavy chain (dark grey). Constant domains and the crystallizable fragment (Fc) are shown in black.

Four IgG1 Fc-containing imaging probes were expressed and purified in this study (Figure 1). These imaging probes consist of an IgG and a single-chain variable fragment (scFv) fused to an Fc domain (scFv-Fc) that bind maltose binding protein (MBP), a single heavy chain variable (VHH) fused to an Fc domain (VHH-Fc) that binds FaDu cells, and an Fc domain. These imaging probes were used to explore how differences in fragment size affects accumulation and binding to immune cells. The antibody and fragments were labeled with IRDye800CW at a labeling ratio between 1 and 2. Imaging probes used for flow cytometry were labeled with IRDye680RD and had a labeling ratio between 1 and 2. Purified and labeled imaging probes were analyzed for size and purity by microcapillary electrophoresis (Figure S1). All probes were within the expected size and were >85% pure.

### 3.2. FaDu Cell Targeted VHH-Fc and MBP Binding scFv-Fc Imaging Probes Accumulate in FaDu Xenografts

**Figure 2:**
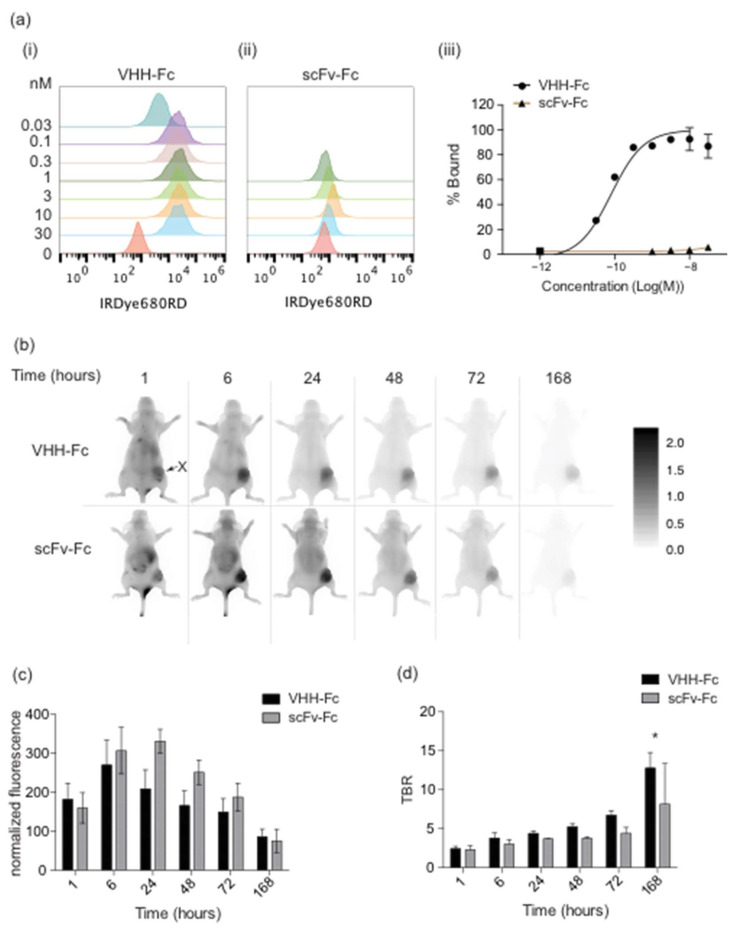
Fc-containing imaging probe binding to FaDu cell lines and xenografts. (**a**) (**i**,**ii**) Histograms and (**iii**) binding curves of VHH-Fc imaging probe, with a VHH domain that binds FaDu cells and scFv-Fc, with an scFv that binds MBP were tested for binding to FaDu cells. (**b**) Fluorescence imaging of the VHH-Fc (top) and scFv-Fc (bottom) probes in CD-1 nude mice bearing FaDu xenografts over time. (**c**) Normalized fluorescence and (**d**) tumor-to-background (TBR) of mouse imaging shown in (**b**). Error bars shown are representative of the standard error of the mean from at least three independent mice. * represents the *p*-value ≤ 0.05. X identifies the location of the xenograft.

The VHH-Fc probe targeting the FaDu cell line interacts with FaDu cells in vitro with an apparent dissociation constant of (K_Dapp_) of 110 ± 7 pM, whereas the scFv-Fc does not bind FaDu cells in vitro (Figure 2a). The IRDye800CW-labeled VHH-Fc and scFv-Fc probes were injected intravenously into mice bearing FaDu xenografts. The scFv-Fc probe accumulated at slightly higher levels and peaked at a later time relative to the VHH-Fc probe; however, there was no statistical difference in tumor accumulation (Figure 2b,c). The VHH-Fc probe signal peaked at 6 h post injection (hpi) with a normalized mean fluorescence intensity (MFI) of 270 ± 64 and the scFv-Fc probe signal peaked at 24 hpi with a normalized MFI value of 330 ± 30 (Figure 2c). There was no significant difference in the tumor-to-background ratio (TBR) between the targeted VHH-Fc and the scFv-Fc probes, except at 168 hpi (*p*-value < 0.05) when the signal was low (MFI < 90 for both constructs). Both probes cleared through the liver (Figure S2) and there was no accumulation in the kidney (Figure S2).

### 3.3. Fc Domain Blocks Tumor Accumulation of FaDu Cell Line-Targeted VHH-Fc Probes

**Figure 3:**
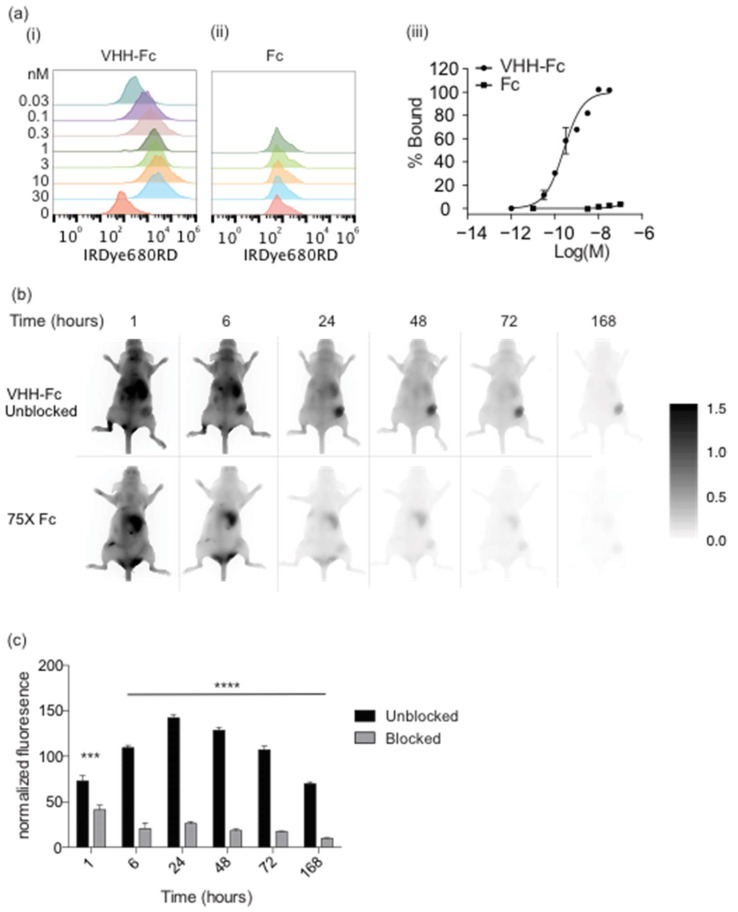
Fc domain blocks accumulation of VHH-Fc imaging probe. (**a**) VHH-Fc and Fc were tested for binding and saturation on MDA-MB-231 cells by titration. (**b**) (top) Fluorescence imaging of IRDye800CW-labeled VHH-Fc probe in CD-1 nude mice bearing MDA-MB-231 xenografts over time. (bottom) Fluorescence imaging of IRDye800CW-labeled VHH-Fc probe in CD-1 nude mice bearing MDA-MB-231 xenografts injected with 75X unlabeled Fc domain. (**c**) Normalized fluorescence of mouse imaging shown in panel (**b**). Error bars represent the standard error of the mean from at least three independent mice and *** represents the *p*-value ≤ 0.001 and **** *p*-value represents ≤ 0.0001.

Since the VHH-Fc and scFv-Fc probes both contain an Fc domain, we determined if the accumulation of these probes in the FaDu xenograft was due to the Fc domain interacting with cells in the xenograft. To do this, we performed a blocking experiment with unlabeled Fc domain. Since the VHH-Fc probe also binds MDA-MB-231 cells (Figure 3a), we used this cell line to rule out artifacts that could be attributed to the FaDu cell line. The VHH-Fc and Fc probes were tested for binding to MDA-MB-231 cells using flow cytometry (Figure 3a). The VHH-Fc probe bound to MDA-MB-231 cells, whereas the Fc probe did not bind. We tested the ability of the VHH-Fc probe to interact with MDA-MB-231 xenografts and analyzed probe accumulation images over time (Figure 3b). The VHH-Fc probe signal peaked at 24 hpi with an MFI of 140 ± 6 (Figure 3b,c). However, when 75-fold excess of unlabeled Fc domain was intravenously injected prior to injection of VHH-Fc probe, accumulation of the VHH-Fc probe in the xenograft was significantly blocked (MFI < 45 at all time points).

### 3.4. Accumulation of Fc-Containing Probes Depends on the Cell Origin of the Xenograft

**Figure 4:**
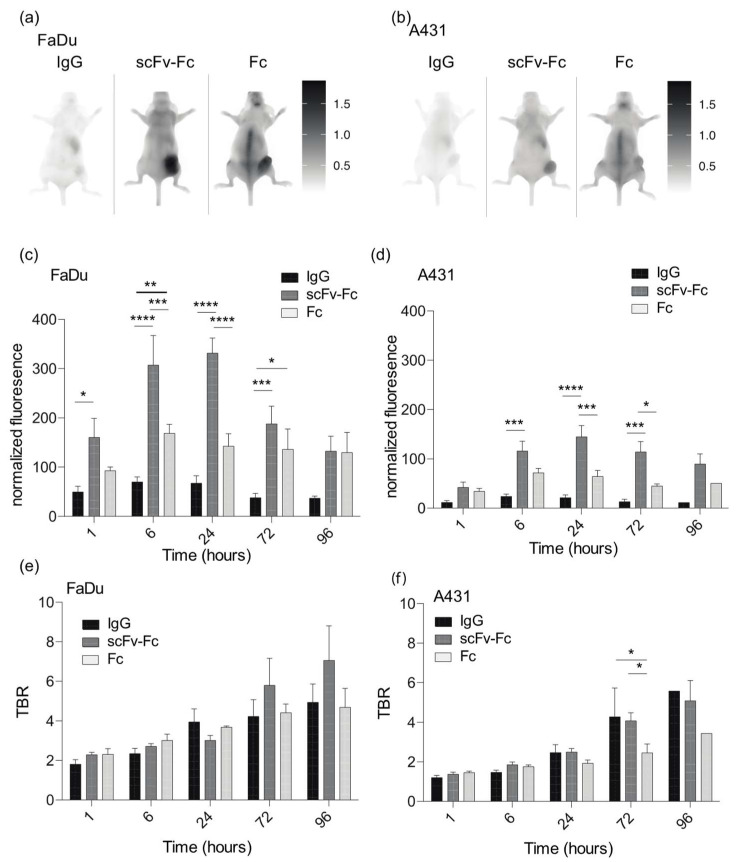
Imaging depends on the Fc-containing imaging probe and the xenograft. (**a**,**b**) IgG, scFv-Fc, and Fc domain probes were injected into CD-1 nude mice bearing FaDu or A431 xenografts and imaged over time with the 24 h image shown. (**c**,**d**) Normalized fluorescence and (**e**,**f**) tumor-to-background ratio (TBR) of mouse imaging over time. Error bars represent the standard error of the mean from at least three independent mice and *p*-value * represents the *p*-value ≤ 0.05, ** represents the *p*-value ≤ 0.01, *** represents the *p*-value ≤ 0.001 and **** represents the *p*-value ≤ 0.0001.

We compared accumulation of Fc-containing probes in xenografts generated from FaDu and A-431 cells. Fc, scFv-Fc, and IgG probes were characterized for binding to A-431 and FaDu cells using flow cytometry (Figure S3). None of these probes bound to these cell lines in vitro. We tested the accumulation of these Fc-containing probes in FaDu and A-431 xenografts in vivo (Figure 4). Accumulation of the smaller Fc-containing probes (scFv-Fc and Fc domain) was higher than the IgG probe. In FaDu xenografts, the scFv-Fc probe was significantly higher than the IgG probe at all time points (*p*-value ≤ 0.05 at 1 hpi, *p*-value ≤ 0.0001 at 6 and 24 hpi and *p*-value ≤ 0.001 at 72 hpi), except at 96 hpi and significantly higher than the Fc domain probe at 6 and 24 hpi (*p*-value ≤ 0.001 and 0.0001, respectively) (Figure 4c). Accumulation of the Fc domain probe in FaDu xenografts was significantly higher than the IgG probe at 6 and 72 hpi (*p*-value ≤ 0.01 and 0.05, respectively) (Figure 4c). Signal from the Fc domain probe peaked at 6 hpi in FaDu xenografts with an MFI of 170 ± 18, whereas the signal of the scFv-Fc probe peaked at 24 hpi with an MFI of 330 ± 30. The IgG probe had little accumulation in both xenografts at all time points (MFI < 70) (Figure 4a,b).

In the A-431 xenograft, the scFv-Fc probe accumulated significantly more than the IgG probe at 6, 24 and 72 hpi (*p*-value ≤ 0.001 at 6 and 72 hpi and *p*-value ≤ 0.0001 at 24 hpi) (Figure 4d) and significantly more than the Fc domain probe at 24 and 72 hpi (*p*-value ≤ 0.001 and 0.05, respectively). The Fc domain probe did not accumulate at high levels in A-431 cells (MFI < 72 at all time points). Accumulation of the scFv-Fc probe in A-431 cells peaked at 24 h with an MFI of 145 ± 22.

The TBR for all probes were similar at each time point in both cell lines (Figure 4b). They all had a TBR around 2 at 1 hpi, which slowly increased to between 4.5 and 7 at 96 hpi. The TBR for A-431 cells was similar; however, the IgG and scFv-Fc probes were significantly higher at 72 hpi compared to the Fc domain probe (*p*-value ≤ 0.05). All three probes cleared through the liver and had no accumulation in the kidney (Figure S4).

### 3.5. Analysis of Fc Domain Binding in Mouse Xenografts and Bone Marrow

**Figure 5:**
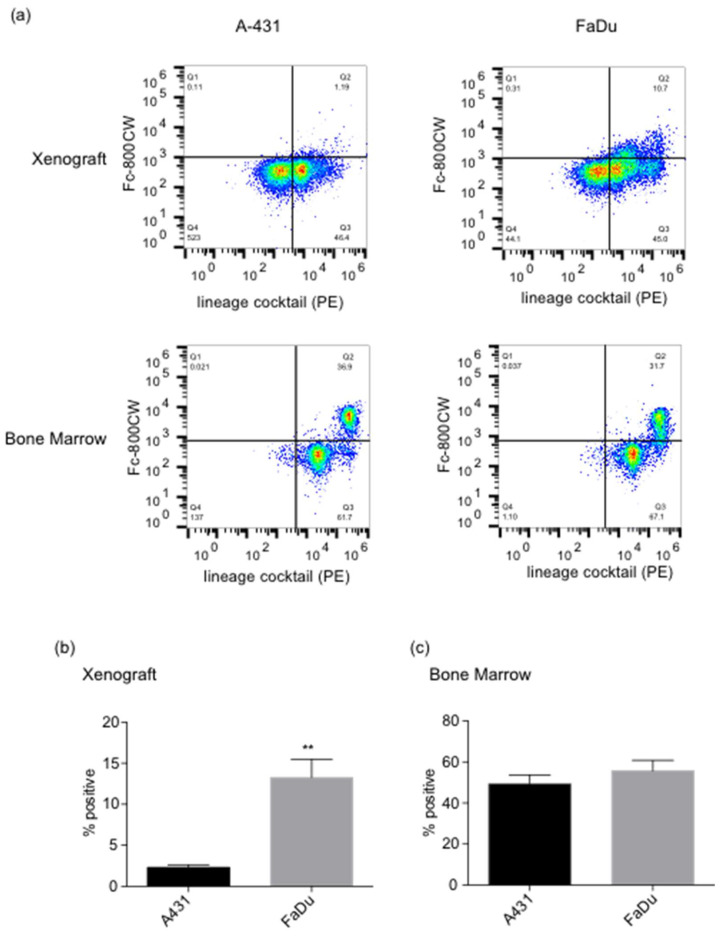
Flow cytometry analysis of cells collected from mouse xenografts and bone marrow. Tumor cells and bone marrow from CD-1 mice with A-431 and FaDu xenografts injected with IRDye800CW-Fc probe were collected, incubated with a lineage marker antibodies, and analyzed by flow cytometry. (**a**) Dot plot of representative flow cytometry with lineage markers and IRDye800CW-Fc probe. (**b**) Percent positive cells for Fc and Fc+ lineage marker in the xenografts. ** indicates *p* ≤ 0.01. (**c**) Percent of positive cells for Fc and Fc+ lineage marker in the bone marrow.

Since injecting an excess of Fc domain blocked the VHH-Fc probe binding to MDA-MB-231 xenografts, we reasoned that the Fc domain could be binding to Fc receptors on tumor-infiltrating immune cells. To determine which mouse cells the Fc domain probe was binding, we intravenously injected the IRDye800CW-Fc domain probe into mice engrafted with A-431 and FaDu cells and characterized its binding to tumor cells from A-431 and FaDu xenografts. Cells from xenografts and bone marrow were collected and analyzed by flow cytometry (Figure 5).

Xenograft cells were stained with lineage markers for differentiated mouse blood cells and approximately half of cells collected stained positive for lineage markers. There were <0.5% IRDye800CW positive cells in the lineage negative gate. The majority of IRDye800CW positive cells were found in the lineage marker positive gate, indicating that the Fc domain probe was not binding tumor cells, but instead binding to mouse blood cells. Cells from the A-431 xenografts showed minimal staining (2.3 ± 0.3%) with the IRDye800CW-Fc domain compared to cells from FaDu xenografts, which showed significantly more (13.3 ± 2.2%, *p* = 0.01) lineage positive cells. Cells collected from the FaDu xenografts were also more positive for the IRDye800CW-Fc domain than the A-431 xenografts. This correlated with imaging studies, where FaDu xenografts accumulated more probe than A431 xenografts. Bone marrow cells taken from mice showed a similar staining pattern between all mice with regard to the lineage marker staining. In this case, almost all bone marrow cells were lineage marker positive (>98%), independent of the origin of the mouse xenograft and 30–40% of bone marrow cells were positive for the IRDye800CW-Fc probe.

### 3.6. Binding of Fc Domain Probe to Peripheral Blood

**Figure 6:**
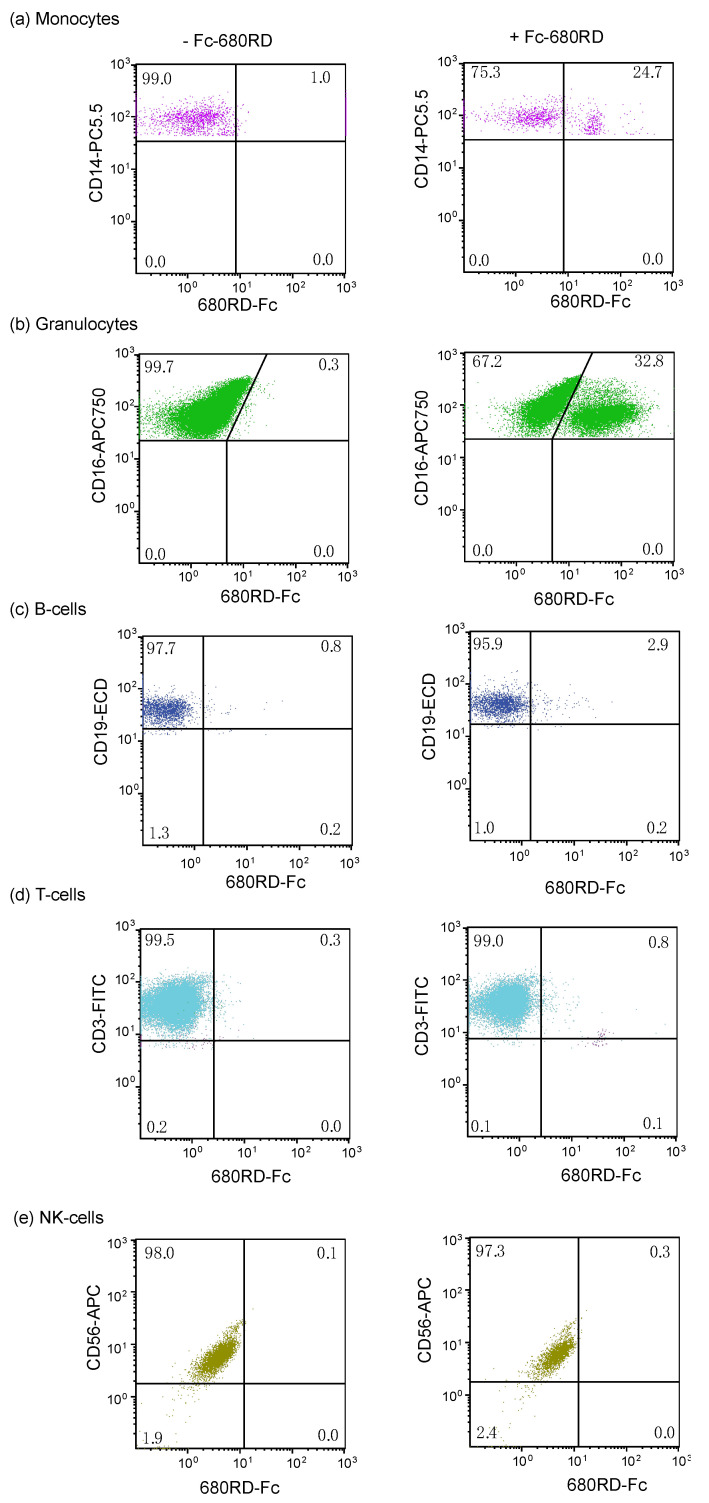
Fc domain probe binds monocytes and granulocytes. Human peripheral blood was incubated with an immune cell panel to differentiates between (**a**) monocytes, (**b**) granulocytes, (**c**) B cells (**d**) T cells and (**e**) natural killer (NK) cells using CD3, CD19, CD14, CD16, CD56, CD45, the IRDye680RD-Fc probe and analyzed by flow cytometry. The percent of cells is shown in each quadrant.

To identify blood cells that interact with the Fc domain probe, we labeled the Fc domain with IRDye680RD and determined human peripheral blood cell types that it interacted with the probe using flow cytometry. We used an immune flow cytometry panel, which identifies immune cells based on surface markers, to identify cell types that the IRDye680RD-Fc probe interacted with (Figure 6). The IRDye680RD-Fc probe did not bind to B cells, T cells or NK cells (Figure 6); however, 25% of monocytes and 33% of granulocytes bound to the IRDye680RD-Fc probe (Figure 6).

**Figure 7:**
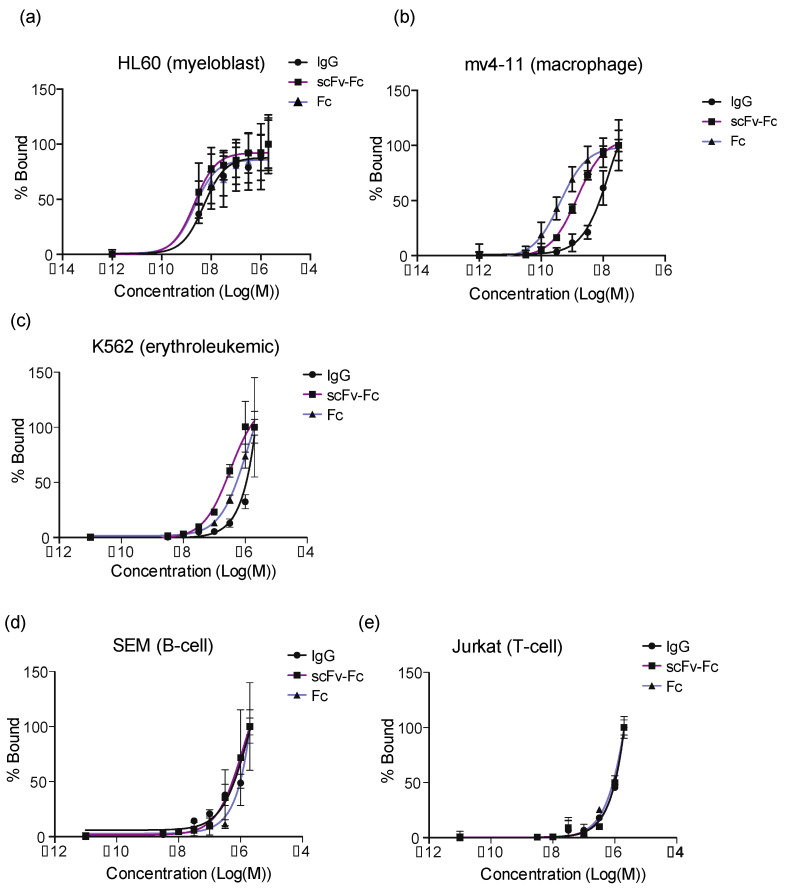
Binding of Fc-containing probes to cell lines using flow cytometry**.** The percentage of IgG, scFv, and Fc domain probes bound to indicated cell lines versus probe concentration. (**a**) HL-60 is a myeloblast cell line that differentiates into granulocytes. (**b**) Mv4-11 is a macrophage cell line. Jurkat is a T-cell line. (**c**) K562 are erythroleukemia cells that can spontaneously differentiate into erythrocytes, granulocyte and monocytes. (**d**) SEM is a B-cell line. (**e**) Jurkat is a T-cell line.

We determined the K_Dapp_ values of IgG, scFv-Fc, and Fc domain probes with cell lines representative of different cell types. Limited binding of probes was observed in the T-cell line (Jurkat) and the B-cell line (SEM). The erythroleukemia cell line (K652) showed weak binding and did not saturate (Figure 7).

The Mv4-11 and HL-60 cell lines, which represent macrophages (monocytes) and cells that spontaneously proliferate to neutrophils (granulocytes) or monocytes [20,21], respectively, bound to the Fc-containing probes with high affinity (Figure 7). The IgG, scFv-Fc, and Fc probes bound to the HL-60 cell line with binding constants of 5 ± 0.3, 2.1 ± 0.1, and 3.6 ± 2.3 nM, respectively, and to mv4-11 with binding constants of 1.7 ± 0.2, 1.5 ± 0.05, and 0.4 ± 0.02 nM, respectively.

## 4. Discussion

Imaging probes that non-invasively monitor immune response can be used to identify infections, tumors, and monitor efficacy and safety of therapies. In this study, we examined imaging properties of Fc-containing probes (IgG, scFv-Fc, and Fc) in mouse cancer xenografts. Both scFv-Fc and Fc probes accumulated in mouse xenografts. The scFv-Fc probe accumulated at higher levels in the tumor than the Fc domain probe, and the IgG showed very low levels of accumulation. The larger size of the scFv-Fc may play a role by increasing circulation time, which leads to a longer half-life, giving it more time to accumulate in the tumor [22]. Interestingly, the IgG showed lower levels of accumulation in the xenograft despite being the largest molecule. This may be due to the IgG having difficulty penetrating the tumor/tumor vasculature [22], or steric hinderance in FcR binding, which is supported by our observation that IgG typically binding the weakest to cell lines we tested. Therefore, attaching an Fc domain to an existing targeting molecule can potentially further increase accumulation if immune cells are near target cells; however, it is difficult to determine whether the probe is binding the target cell, immune cells, or both, which can cause accumulation in tissues where they may not be expected to accumulate. This can be exploited to design imaging probes that accumulate at higher levels in tissues that contain target and immune cells.

The role of the Fc domain in the probe accumulation can be tested by including a blocking dose of an Fc domain control in imaging experiments. In addition to tumor uptake, we observed accumulation of Fc-containing probes in the backbone, under the armpits, in the neck and leg bones of mice, which are locations of lymph nodes and large bones containing bone marrow. Lymph nodes and bone marrow contain immune cells and hemopoietic cells, both of which express Fc receptors. Accumulation of the scFv-Fc and IgG was not visible in these areas. We showed that uptake of the Fc domain probe in these areas was likely due to the Fc domain binding to immune cells in the tumor environment. Using human PBMCs, we showed that the Fc domain alone can bind to granulocytes and monocytes. This is consistent with the literature which shows that certain granulocytes and monocytes have FcγRI (CD64), FcγRII (CD32), and FcγRII (CD16), all of which bind to IgG [23].

It is well established that certain blood cells contain Fc receptors and that they are difficult to analyze by flow cytometry and immuno-histochemistry due to non-specific antibody binding. Often an Fc blocking buffer is used to block these interactions. (Fab)_2_ or other non-Fc-containing antibody fragments have been developed to avoid Fc binding. Here, we show how the Fc binding can be exploited for use as an imaging probe to non-invasively image innate immune responses.

Currently, many cancer diagnostic and therapeutic monitoring assays rely on biopsies and histology. There is potential to use imaging probes to analyze the tumor microenvironment using targeted molecular imaging probes. For example, in solid tumors immune cells are involved in tumor infiltration, including T and B lymphocytes, natural killer cells, NK-T cells, dendritic cells, macrophages, neutrophils, eosinophils, and mast cells [24]. Immune cell infiltration of cells varies between different cancers and can potentially be used as a predictor of disease and therapeutic outcome. Neutrophils and macrophages are associated with poor prognosis due to their tumor promoting activities, both of which bind Fc domains. Most tumors have immunosuppressive macrophages, which contribute to tumor progression [25]. Increased levels of neutrophils in tumor tissues (including but not limited to hepatocellular, esophageal squamous cell carcinoma, melanoma, colorectal cancer, pulmonary adenocarcinoma, and renal cell carcinoma) is correlated with poor prognosis [4,5,26,27,28,29]. Imaging probes, such as the Fc domain, could be used to identify levels of neutrophils and macrophages. Non-invasive imaging can be used to determine levels of innate immune infiltration and potentially aid in identifying disease states. The benefit of monitoring innate immune response extends beyond cancer and would be useful for a number of other diseases such as cardiovascular diseases, inflammation, ischemia, and multiple sclerosis [30,31].

Monitoring and predicting therapeutic efficacy are other important areas that would benefit from non-invasive imaging probes. Most therapies are expensive and prone to side effects, which can lead to non-compliance. Predicting and monitoring if a therapy will work or is working would reduce costs and avoid unnecessary treatments and side effects. Positive feedback to the patient could encourage them to continue therapy, since it can take weeks to months and invasive procedures to determine if a therapy is working. With imaging, it could be monitored over time and efficacy could potentially be determined sooner. For example, neutrophil infiltration in metastatic colorectal cancer not only indicates lower survival rates but also shows negative effects against bevacizumab treatment [5]. Knowing this would prevent these patients from adverse events associated with bevacizumab treatments. Macrophages have been tracked to monitor the inhibitory effect of dexamethasone to macrophage migration [32], which could be useful in monitoring recruitment or disappearance of macrophages before and during other therapies.

People suffering from inflammatory diseases such as rheumatoid arthritis or encephalomyelitis could also benefit from imaging with the Fc by providing earlier diagnosis and a non-invasive monitoring tool. Rheumatoid arthritis currently is diagnosed, managed, and monitored by radiography of the hands or feet [33]. This type of imaging is not sensitive enough to detect early disease and can only diagnose rheumatoid arthritis once there is cortical bone damage. Earlier diagnosis could occur with imaging agents such as the Fc since rheumatoid arthritis is characterized by persistent inflammation [33]. Acute disseminated encephalomyelitis is typically diagnosed by neuroimaging with MRI or CT [34]. Lesions in the brain can be detected and while these lesions can be reversible, recurrence can occur. Monitoring infiltration of macrophages which occur early on in these cases would be useful to identify reoccurrence before damage to the brain has occurred. A lumbar puncture can be performed to detect infection with cerebral spinal fluid; however, this method is very invasive, especially when performed multiple times.

The Fc imaging probe is useful for determining immune cell infiltration in the tumor environment or for identifying sites of inflammation. While establishing xenografts in athymic CD-1 nude mice, which lack T cells, we observed that some cell lines have a higher rate of rejection (FaDu has a higher rate of rejection versus A-431 for example). Xenograft rejection has been previously shown to be due to infiltrating macrophages and T cells [35]. Failure to establish xenografts correlates with the non-specific uptake of the Fc domain. For example, FaDu xenografts had more Fc probe accumulation and were more difficult to engraft than A-431 xenografts, which implies there are more immune cells in the FaDu xenografts which can prevent the xenograft from growing in some mice. In a similar way, the Fc probe could be used to monitor therapies, for example, if a therapy is causing an immune reaction the Fc probe will accumulate which could indicate if the therapy is working or not. The Fc imaging probe is also useful to study other types of inflammatory diseases and infections. For example, Fischman et al. previously showed that the Fc domain can be used to image infection in rats caused by *Escherichia coli* when it was labeled with indium-111 [36]. The Fc domain was compared to a non-specific IgG, Fab, and single subunit of an Fc domain and the Fc domain showed the best imaging properties when comparing liver and kidney accumulation, target-to-background ratio, and percent residual activity. We are currently working to fully characterize the Fc as an imaging agent to be used to study the dynamics of the innate immune system and to be used as a PET imaging agent.

## 5. Conclusions

We report for the first time the use of the hIgG Fc domain for imaging of immune cells in xenograft tumors in mice. The IRDye800CW-labeled Fc domains localize to tumors that are visible by fluorescent imaging. Localization of the Fc domain in xenografts seems to be due to binding tumor-associated immune cells and signal dependent on the cell line used to develop xenografts. The Fc domain can bind to different human immune cell types. This work represents a novel option to study innate immune response. Future studies will focus on fully characterizing the Fc domain for PET imaging.

## 6. Patents

The work in this manuscript has been filed for a US Provisional Patent, ‘Fc Domain imaging probes and methods of use thereof’, United States Provisional Application No. 63/243,411, (2021).

## Data Availability

Data is contained within the article or Supplementary Material.

## Supplementary Materials

The following are available online at https://www.mdpi.com/article/10.3390/cancers14020300/s1, Figure S1: Purity and molecular weight analysis of expressed and labeled proteins; Figure S2: Liver-to-background and kidney-to-background ratios for VHH-Fc and scFv-Fc; Figure S3: Control IgG, scFv-Fc and Fc do not bind to FaDu or A-431 cells; Figure S4: Liver-to-background and kidney-to-background ratios for IgG, scFv-Fc, and Fc.
