# Supplementary material for: Imaging Immune Cells Using Fc Domain Probes in Mouse Cancer Xenograft Models
Source: Cancers (Basel). 2022 Jan 8;14(2):300. doi: 10.3390/cancers14020300 (PMC8773629; doi:10.3390/cancers14020300)
Supplement: Supplementary file 1 [file cancers-14-00300-s001.zip › cancers-1520691-supplementary.pdf]

cancers MDPI

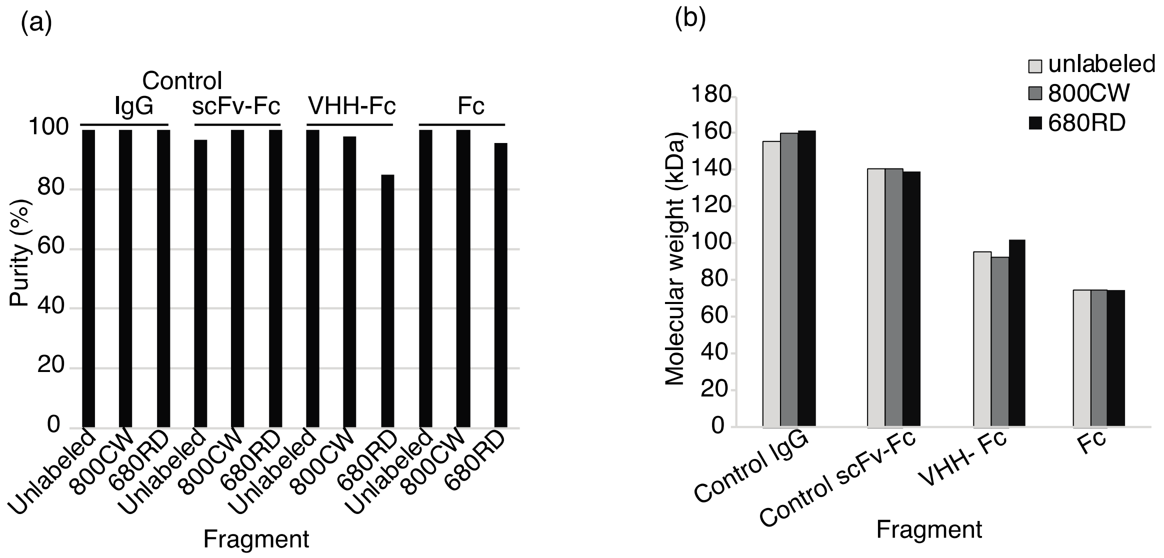

Figure S1. Purity and molecular weight analysis of expressed and labeled proteins. Proteins used in this study were expressed in expi293 cells and purified with a MabSelect SuRe purification column. The proteins were labeled with IRDye680RD or IRDye800CW and analysed for (**a**) purity and (**b**) molecular weight.

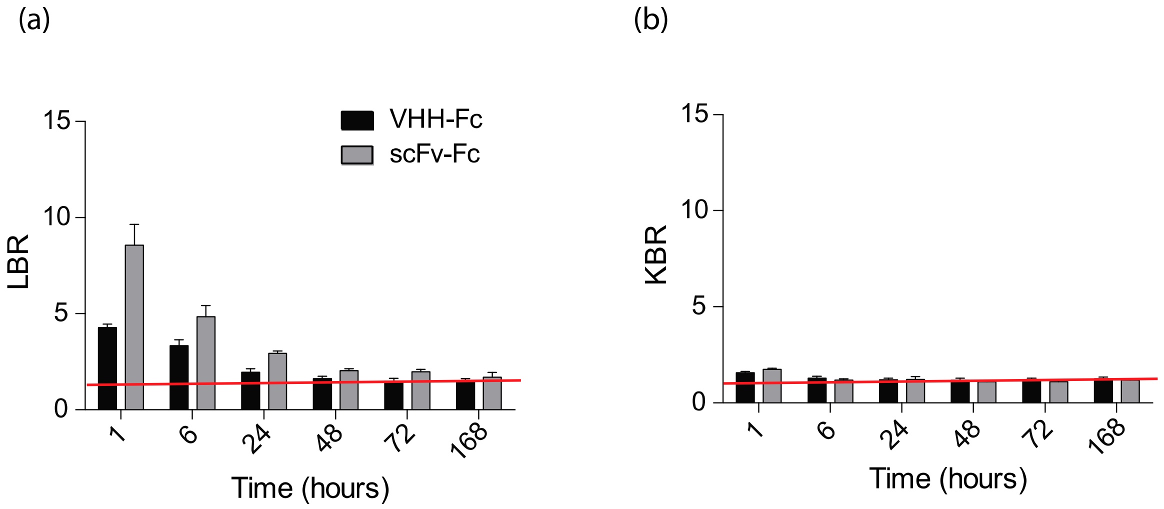

Figure S2. Liver-to-background and kidney-to-background ratios for VHH-Fc and scFv-Fc. CD-1 nude mice bearing FaDu xenografts were injected with targeted VHH-Fc or scFv-Fc and imaged over time. The (**a**) liver-to-background ratio (LBR) and (**b**) kidney-to-background ratio (KBR) were quantitated. Red line drawn at a TBR of 1. Error bars show the standard error of mean and representative of at least three mice.

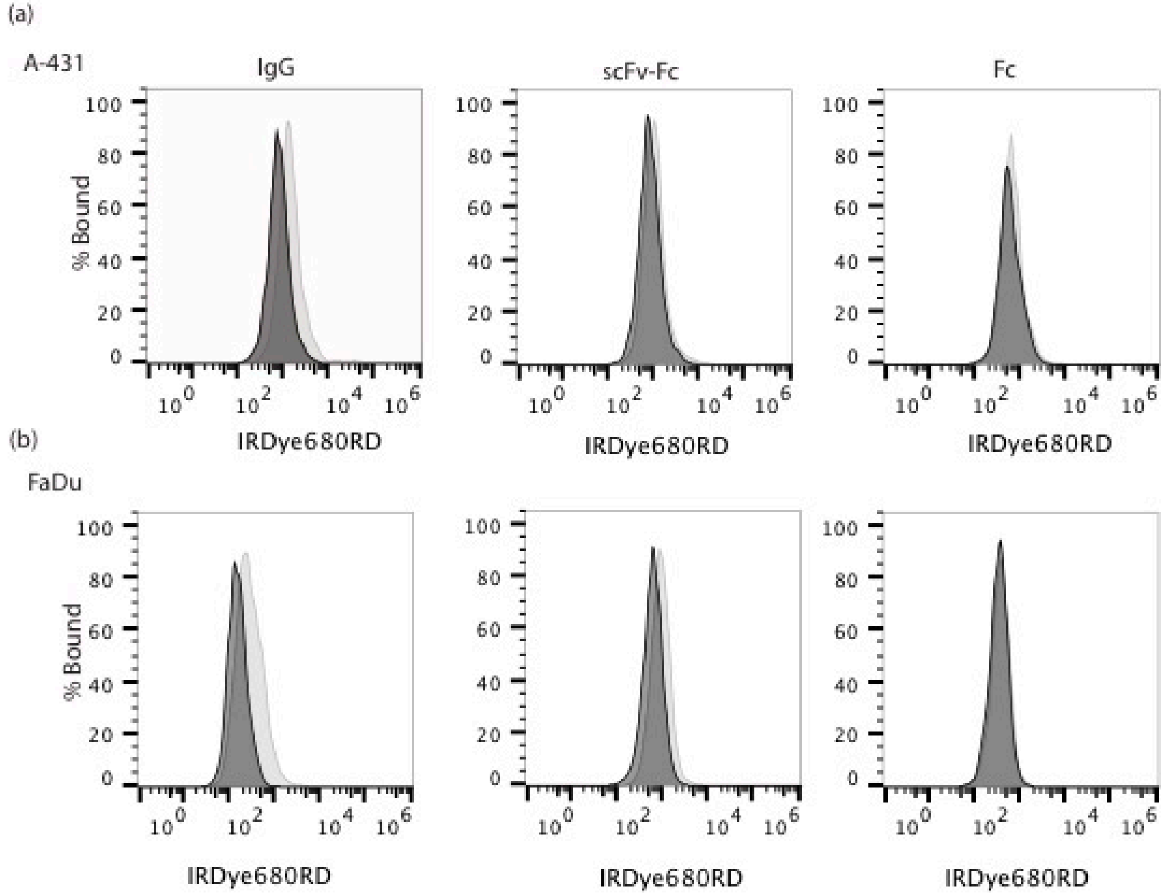

Figure S3. IgG, scFv-Fc and Fc do not bind to FaDu or A-431 cells. **IRDye680RD-labeled** IgG, scFv-Fc or Fc were mixed at a concentration of 100 nM with (**a**) A-431 or (**b**) FaDu cells and analyzed by flow cytometry.

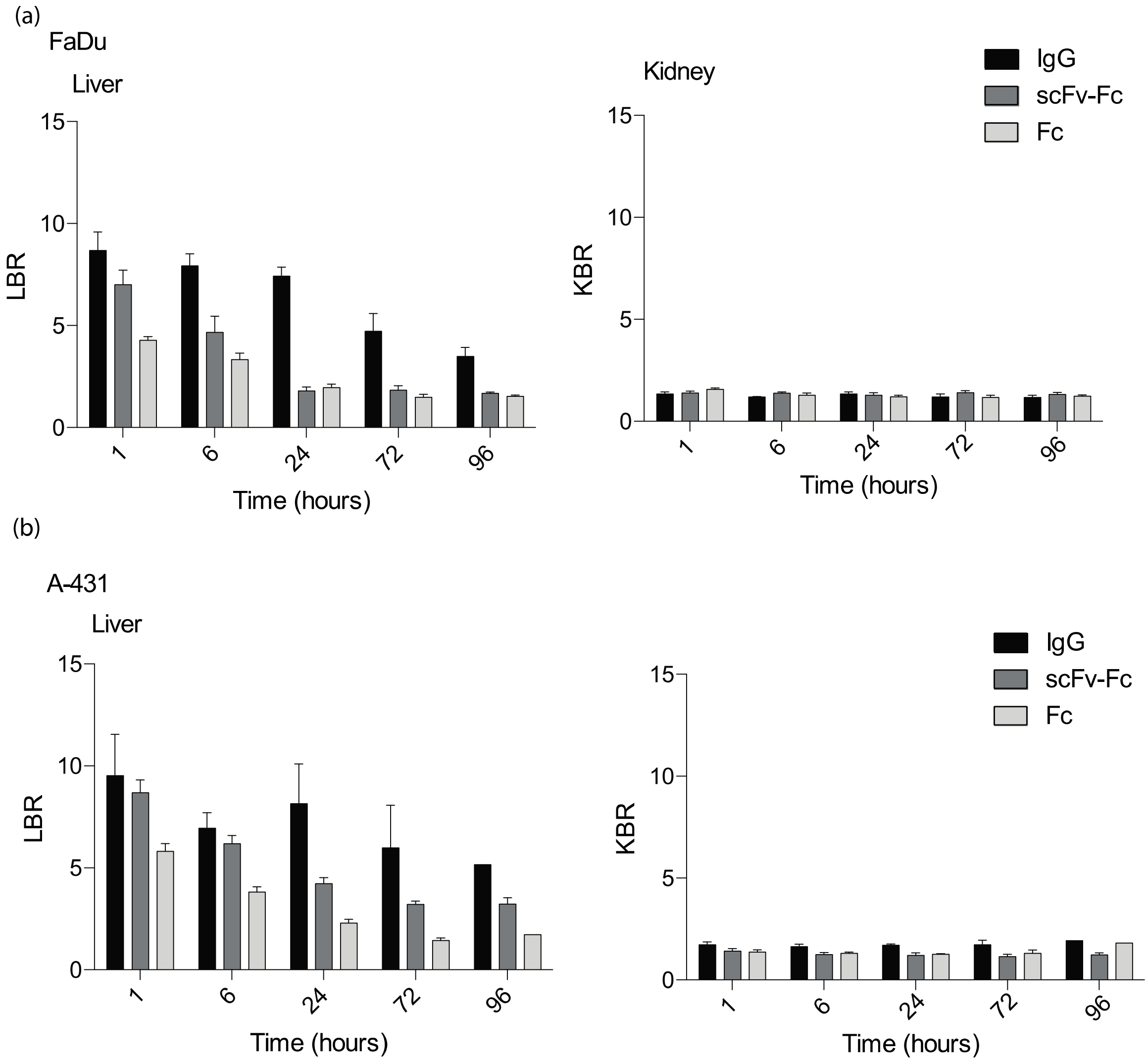

Figure S4. Liver-to-background and kidney-to-background ratios for IgG, scFv-Fc, and Fc. CD-1 nude mice were injected with IgG, scFv-Fc or Fc1 in mice bearing (**a**) FaDu or (**b**) A-431 xenografts and imaged over time. The liver-to-background ratio (LBR) and kidney-to-background ratio (KBR) were quantitated. Error bars show the standard error of mean and representative of at least three mice.
